# 

**Published:** 1899-02-25

**Authors:** 


					THE hospital.' " The "standard ofjRhighest ^purity."?Lancet* Feb. 25th, 1899
~caiassr8 t H ? CAD?
ABSOLUTELY PURE. ^ NO DRUGS OR ALKALIESIUSED.
mntcH hosrutax
648. Vol. XXY. ffSSS?i S^xtbday,
Feb. 25th, 1899.
[-SubKcriptionTMms.] pRICE TWOPENCE.

				

